# The Prevalence of Abdominal Obesity and Its Correlates among the Adults in Dodoma Region, Tanzania: A Community-Based Cross-Sectional Study

**DOI:** 10.1155/2018/6123156

**Published:** 2018-10-17

**Authors:** Mariam John Munyogwa, Abdalla Hussein Mtumwa

**Affiliations:** ^1^Department of Public Health, University of Dodoma, P.O. Box 395, Dodoma, Tanzania; ^2^Department of Statistics, University of Dodoma, P.O. Box 338, Dodoma, Tanzania

## Abstract

**Introduction:**

Overweight and obesity are a threat to the public health following their association with noncommunicable diseases, such as type 2 diabetes mellitus, cardiovascular disease, and some cancers. Despite this fact, the information on overweight and obesity, particularly in most developing countries, is still scarce to address the problem. This article partly addresses the gap through the findings of a cross-sectional survey that was conducted in Dodoma Region, Central Tanzania, to determine the prevalence and correlates of abdominal obesity among adults.

**Methods:**

Using a community-based cross-sectional survey, data were generated from the participants who aged 18 years and above. Simple random sampling and Kish selection table techniques were used to get the sample who responded through a face-to-face-administered questionnaire. Waist circumference was measured using the guideline of the WHO protocol of measuring waist and hip circumference. Abdominal obesity is defined as a condition with waist circumference >102 cm for men and >88 cm for women. Prevalence was computed with a 95% confidence interval. Simple and multiple logistic regression models were fitted to identify the risk factors associated with abdominal obesity.

**Results:**

A total of 840 respondents took part in the study. The overall prevalence of abdominal obesity was found to be 24.88% (209/840). The prevalence of abdominal obesity was significantly higher among women than men (35.14% vs. 6.89%, *p* < 0.0001) and higher among urban dwellers (33.56%) than their rural counterparts (15.56%). Correlates of abdominal obesity was found to be gender, marital status, place of residence, age, education level, and the time used in watching television.

**Conclusion:**

This study revealed a high prevalence of abdominal obesity among the people living in the Dodoma Region. Increased age, urban residence, more time spent on television, less walking per day, and being ever married were all associated with having abdominal obesity in this population.

## 1. Introduction

The number of people with both generalized and abdominal obesity has been increasing worldwide [1–4]. The prevalence of obesity has almost doubled over the last 30 years globally [5, 6]. According to the World Health Organization report of 2015, more than 1.9 billion adults (18 years and older) were overweight and, among them, over 650 million were obese [6]. Overweight and obesity are a threat to the public health as they increase the likelihood of noncommunicable diseases, such as diabetes, hypertension, coronary heart disease, stroke, certain cancers, obstructive sleep apnoea, and osteoarthritis and negatively affect reproductive performance [6–9].

The fundamental cause of overweight and obesity is the energy imbalance between calories consumed and calories expended [10]. Globally, there has been an increased intake of energy-dense foods that are high in fat and increase in physical inactivity due to the increasingly sedentary nature of many forms, such as spending much time on TV, changing modes of transportation, the increasing urbanization, and so on [11].

Over the past years, overweight and obesity were considered the problem of the high-income countries; however, for the last three decades, the number of people with overweight and obesity had risen significantly in the low- and middle-income countries (LMIC), and particularly in the urban settings [12]. Research from African countries shows that overweight and obesity have been increasing rapidly over the past twenty-five years, particularly in the urban settings [12]. If this is not controlled, it may take epidemic proportions in the very near future [13].

In Tanzania, the prevalence of overweight and obesity have been reported higher, particularly in urban areas of the country [14–22]. The findings from the previous studies show that the prevalence of overweight and obesity had increased significantly since the 1980s, and the trend shows the prevalence has been doubling after every decade since the 1990s [6, 12, 15, 20, 22]. However, many previous studies were conducted in cities and the highly urbanized areas of the country, particularly in Dar es Salaam city [14, 15, 17–21]. This has resulted in the inadequacy of overweight and obesity information from other areas of the country. Needless to say, Tanzania is a large and geographically diverse country [23]. There is a need for this information from all areas of the country to help the health sector to equally plan and prioritize health programmes in the community. It had been emphasized earlier that local health information is very important for a country like Tanzania [24]. Another limitation of the previous studies conducted in Tanzania is that they had much of their focus on generalized obesity [14–22]. While this has been their focus, the evidence available shows that abdominal/central obesity is the best predictor of diseases than the generalized obesity [25, 26]. This study observed this research opportunity by looking at the prevalence of abdominal obesity and its correlates among adults in Dodoma Region, Central Tanzania.

## 2. Methodology

### 2.1. Study Area

Administratively, the United Republic of Tanzania is divided into 30 regions (i.e., 25 in Tanzania mainland and 5 in Tanzania Zanzibar) [23]. The regions are further divided into districts. The districts are further divided into wards and wards into streets and villages in urban and rural settings, respectively.

This study was conducted in one of the regions of the country, namely, Dodoma. Dodoma Region is found at the centre of the country, and it is a semiarid area of Tanzania. The Region is the 12^th^ largest in the country and covers an area equivalent to 5% of the total area of Tanzania mainland [27]. Administratively, the Region has seven districts with a total population of 2.08 million and annual growth rate of 2.1 [23]. Ethnic groups are composed of Bantu-speaking people. Indigenous groups include Gogo, Rangi, Sandawe, Nguru, Zigua, Kaguru, Wambulu, and Wasagara who make almost three quarters of the total population. However, due to urbanization, many societies from various areas inside and outside the country are represented in the Region [27].

### 2.2. Study Design and Study Population

This study is a quantitative cross-sectional survey. The survey was conducted from January 2014 to January 2015. Study population involved adults aged 18 years and above living in the Dodoma Region.

### 2.3. Sampling and Sampling Procedures

A multistage sampling technique was used to get a sample of 840 people. Wards were first stratified into two strata (rural and urban). Then, from each stratum, the representative wards were selected using simple random sampling methods. From selected wards, the representative streets or villages and households were selected by simple random sampling methods. From each of the selected household, a Kish selection table was used to select one participant. Probability proportional to size allocation was used to distribute subjects in the wards and street or villages.

### 2.4. Data Collection and Study Procedure

Participants' demographic and lifestyle information were obtained through face-to-face interviews using a standard pretested questionnaire that was adopted from WHO steps for surveillance of noncommunicable diseases and modified for the study environment. Waist circumference was measured using the guideline of the WHO protocol for measuring waist and hip circumference [28]. Waist circumference was taken approximately at the midpoint between the lower margin of the last palpable rib and the top of the iliac crest. The measurements were done in triplicates, and the average was used to represent the WC of an individual. Abdominal obesity was defined as a condition with waist circumference greater than 102 cm for men and greater than 88 cm for women [28].

### 2.5. Data Analysis

Data were analyzed using the Statistical Analysis System (SAS) 9.4. Preliminary data analysis included descriptive statistics, i.e., means and standard deviations for continuous variables and frequencies and percentages for categorical variables which describe the population characteristics. To identify the correlates of abdominal obesity, both simple and multiple logistic regression models were fitted. Simple logistic regression models were fitted to evaluate each independent variable for their unadjusted association with abdominal obesity. All independent variables with *p* value less than or equal to 0.25 in simple logistic regression models were included in the multiple logistic regression model to evaluate factors associated with abdominal obesity among adults. All probabilities were two-tailed, and independent variables with *p* values <0.05 were regarded as significant.

### 2.6. Ethical Considerations

The authorization to perform the study was obtained from the University of Dodoma Ethical Review Committee Board. The study objectives were clearly explained to the participants who gave their informed consent. Data were processed anonymously and confidentially. Researchers kept their promise to widely disseminate the results of the study within health sector partners and the community.

## 3. Results

### 3.1. General Characteristics of the Study Population

The general characteristics of the study population stratified by gender are illustrated in Table 1. A total of 840 participants were involved in this study, whereby 305 (36.31%) were men and 535 (63.69%) were women. The overall mean age of the study participants was 46.01 ± 15.72 years. Men were significantly older than women (47.82 ± 16.80 versus 44.97 ± 14.99, *p*=0.0117, respectively). The number of people living in urban areas was 435 (51.79%), while that of people from the rural area was 405 (48.21%). A majority of the participants were married (67.62%) and the percentage of married subjects was significantly higher among men (80.66%) than women (60.19%). Most (60.12%) of the study population had the primary education level. Significantly more men (29.84%) than women (20%) had the secondary or higher level of education. Forty-five percent of the study participants were peasants while men recorded a higher employed rate of 39.34% than women (37.20%). The proportion of study population who report to be smoking were 20.12% with significantly much higher proportion of men than women (45.25% vs. 5.79%, *p* < 0.0001) (Table 1).

### 3.2. Prevalence of Abdominal Obesity

Table 2 shows the distribution of abdominal obesity according to the selected characteristics of the study participants. The overall prevalence of abdominal obesity was 24.88% (209). The prevalence of abdominal obesity was significantly higher among women than men (35.14% versus 6.89%, *p* < 0.0001) and among urban dwellers (33.56%) than rural dwellers (15.56%). The participants who were never married had a lower prevalence of abdominal obesity (12%) compared to other groups of marital status. The prevalence of abdominal obesity was higher among the participants within the age group of 41–50 (32.61%) and lower among those with the age group 18–30 years (12.96%). With respect to the education level, the prevalence of obesity was higher among those with secondary or higher education (30.81%) compared to those with the primary education (23.76%) and those who never attended formal education (20.44%). The prevalence of abdominal obesity was reported to be significantly higher among nonsmokers as compared to the current smokers (29.21% versus 7.69%, *p* < 0.0001). The prevalence was significantly higher among participants who consumed three meals or more (26.16%) than those who consumed less (21.20%) but not statistically significant.

In this study, it was noted that the participants with relatively higher average walking distance per day had lower prevalence of abdominal obesity compared to participants with relatively lower average walking distance per day. For instance, the prevalence of abdominal obesity among the participants who walked less than 0.5 km and 0.5–1 km per day was 31.68% and 27.21%, respectively. Additionally, the lowest prevalence of abdominal obesity was noted among participants who walked more than 5 km per day (17.26%). Regarding the time spent on television, the results showed that the subjects who did not spend any time on television had lower prevalence of abdominal obesity (18.61%) as compared to those who frequently watch television.

The results of a simple logistic regression model show that women had significantly greater odds of having abdominal obesity than men (OR = 7.33; 95% CI: 4.55, 11.81). Likewise, participants who were married (OR = 2.52, 95% CI: 1.40, 4.55), divorced/separated (OR = 3.49, 95% CI: 1.58, 7.69) and widowed (OR = 3.51, 95% CI: 1.75, 7.07) had significantly higher odds of having abdominal obesity than participants who never married.

The odds of having abdominal obesity were almost three or more times among participants living in urban areas than those living in rural areas (OR = 2.74, 95% CI: 1.96, 3.83). Older participants had higher odds of having abdominal obesity 41–50 years (OR = 3.25, 95%, CI: 1.87, 5.65) than young participants, 18–30 years. Although the level of education was not statistically significantly associated with abdominal obesity (*p*=0.0651), the participants with secondary or higher levels of education had higher odds of having abdominal obesity (OR = 1.73; 95% CI: 1.04, 2.90) than the participants with no formal education. With regard to occupation, students (OR = 0.28; 95% CI: 0.10, 0.82) and peasants (OR = 0.56; 95% CI: 0.40, 0.78) were significantly less likely to have abdominal obesity than employees. Those who were smoking (OR = 0.2; 95% CI: 0.11, 0.36) were significantly less likely to have abdominal obesity than nonsmokers. Another correlate of abdominal obesity was the walk distance per day (*p*=0.0142). The odds of having abdominal obesity were significantly higher among participants who walk less than 0.5 km compared to those who walk more than 5 km per day (OR = 1.79; 95% CI: 1.12, 2.88). Similarly, participants who reported to walk a distance of 0.5–1 km per day had 2 times odds of being obese than those who walk more than 5 km per day (OR = 2.22; 95% CI: 1.25, 3.97). The time spent on watching television was also significantly associated with abdominal obesity (*p* < 0.0001) whereby the odds of having abdominal obesity were higher among those who spent some time watching TV than those who never watch television. On the other hand, the number of meals taken per day (*p*=0.1459) was not a significant correlate of abdominal obesity among this study population.

### 3.3. Correlates of Abdominal Obesity among Adults Aged 18 Years and Older

The results of multiple logistic regression model analysis showed that occupation (*p*=0.1854), smoking status (*p*=0.0691), and walking distance per day (*p*=0.2830) were not significantly associated with abdominal obesity. On the other hand, gender (*p* < 0.0001), marital status (*p*=0.0141), place of residence (*p*=0.0003), age (*p*=0.0037), education level (*p*=0.0154), and watching television (*p*=0.0165) were significantly associated with abdominal obesity (Table 2). Women had greater odds of having abdominal obesity than men (AOR = 8.21; 95% CI: 6.44, 19.52). The odds of having abdominal obesity were significantly higher among married (AOR = 3.31; 95% CI: 1.59, 6.92) and divorced/separated (AOR = 3.08; 95% CI: 1.19, 7.96) than those participants who never got married. Likewise, the widowed had significantly greater odds of having abdominal obesity than the subjects who had never married (AOR = 2.65, 95% CI: 1.08, 6.48). The participants from urban areas had two and more times odds of having abdominal obesity than those from rural areas (AOR = 2.17, 95% CI: 1.42, 3.31). The odds of having abdominal obesity were higher among adults who aged 31 years and above as than those aged below 31 years. Regarding the education level, the results of the multiple regression model revealed that the odds of having abdominal obesity was almost 3 times (AOR = 2.57; 95% CI: 1.29, 5.11) higher among participants with secondary and higher levels of education than that of participants who never attended school. With respect to watching television, the results show that the participants who spent some time watching television were more likely to have abdominal obesity than those who never watch television. It was noted that participants who spent less than 2 hours watching television had the highest odds of having abdominal obesity than other groups from this category (AOR = 1.99; 95% CI: 1.23, 3.24).

## 4. Discussion

This study found a high prevalence of abdominal obesity among adults aged 18 years and above who are currently living in the Dodoma Region. This could be as a result of the increasing urbanization in this Region. It had been demonstrated in previous studies conducted in Tanzania that the prevalence of overweight and obesity was higher in urban areas [7, 20, 29]. Similar findings had been reported from other countries worldwide [1, 2, 4].

Consistent with the previous studies [1, 4, 14, 20, 30], this study found that women were more likely to have central obesity than males. Physiological differences between male and female sex [31], types of daily activities, and cultural issues among sex could be among the possible explanation for this result [32]. Moreover, the differences among males and females in residence type, educational level, occupation types, and average walking distance that were observed in this study could also be attributed to this finding. However, the difference in prevalence between males and females noted from this study is very large; hence, we recommend further studies on this area.

Similar to the previous findings [4, 7, 20, 33–37], our study found a higher prevalence of abdominal obesity in urban population than in the rural counterpart. The prevalence of abdominal obesity in urban was almost twice as that of the rural, and people living in urban areas had more than two chances of having abdominal obesity than those living in rural areas. Urban life is more characterized of unhealthy food such as high energy food and sedentary lifestyles such as reduced walking due to the availability of motorized transport, and so on[37–41], that predispose people into the risk of becoming obese. In the Dodoma Region, the number of commuter buses had increased by 185.2% in a period of seven years from 2003 to 2010 in the Dodoma Region [42]. This might suggest that people living in Dodoma are becoming more exposed to motorized transport, thus making them have less physical exercises, such as walking. Alongside this, improved infrastructure and increased employment opportunities have increased the availability of commodities and services, such as food supplies, fast food services, sugary/soft drinks beverages, food groceries, and so on, to be easy in the Region, whereby some of these services such as fast food and the like increase the exposure of people to unhealthy eating and lifestyles that might predispose people into developing obesity. Likewise, the increased employment opportunities in the Region had improved the economic status of people in the Region enabling them to access the services available in the Region.

In this study, the risk of abdominal obesity is observed to be increased significantly with age. This is in agreement with the previous studies [1, 2, 4, 43–45]. The possible explanation for this could be reduced basal metabolism and reduced physical activities due to age.

In this study, it was noted that being ever married was a risk factor of having abdominal obesity. The reasons for this could not be established by the present study. Similar findings have been reported by the study that conducted in Kinondoni District, Tanzania [19]. Contrary to previous studies [19], this study found that lower education than secondary school was protective for abdominal obesity. This might be due the reasons explained earlier by the previous studies that obesity in most developing countries is regarded as a symbol of power, wealth, and beauty, and hence, it is very common for the people with high social status [42]. Moreover, in our country, people with secondary or higher education are more likely to have formal employment than those with the primary or lower education level. Previous studies have reported that being employed is a risk factor for obesity. However, in this study, employment was found to be a risk factor for abdominal obesity only during univariate analysis. Similar to the previous studies [46], the present study found that, watching television was associated with abdominal obesity. This could imply that sedentary lifestyles, i.e., using television, computers, and the like, occupy people's time of doing physical activities, such as walking, gardening, and so on, which would prevent them from developing obesity and overweight.

## 5. Conclusion

This study revealed a high prevalence of abdominal obesity among people living in the Dodoma Region. The correlates of abdominal obesity found in this study were increased age, sex, area of residence, time spent on television, less walk, and marital status. These results may, therefore, call for respective stakeholders to plan appropriate preventive measures that will prevent further health problems that are associated with obesity.

## Figures and Tables

**Table 1:** General characteristics of the study population stratified by gender.

Variable	Total (*n* = 840)	Men (*n* = 305)	Women (*n* = 535)	*p* value
Age (mean ± SD)	46.01 ± 15.72	47.82 ± 16.80	44.97 ± 14.99	0.0117
WC (mean ± SD)	82.21± 13.72	81.12 ± 13.46	82.83 ± 13.83	0.0819
*Marital status*				<0.0001
Married	568 (67.62%)	246 (80.66%)	322 (60.19%)	
Divorced/separated	56 (6.67%)	9 (2.95%)	47 (8.79%)	
Widowed	99 (11.79%)	5 (1.64%)	94 (17.57%)	
Never married	117 (13.93%)	45 (14.75%)	72 (13.46%)	
*Place of residence*				0.0630
Urban	435 (51.79%)	145 (47.54%)	290 (54.21%)	
Rural	405 (48.21%)	160 (52.46%)	245 (45.79%)	
*Age (years)*				0.1070
18–30	162 (19.29%)	59 (19.34%)	103 (19.25%)	
31–40	169 (20.12%)	50 (16.39%)	119 (22.24%)	
41–50	184 (21.90%)	64 (20.98%)	120 (22.43%)	
51+	325 (38.69%)	132 (43.28%)	193 (36.07%)	
*Education level*				<0.0001
Never attended school	137 (16.31%)	29 (9.51%)	108 (20.19%)	
Primary education	505 (60.12%)	185 (60.66%)	320 (59.81%)	
Secondary or higher	198 (23.57%)	91 (29.84%)	107 (20.00%)	
*Occupation*				
Employed	319 (37.98%)	120 (39.34%)	199 (37.20%)	0.0099
Students	35 (4.17%)	16 (5.25%)	19 (3.55%)	
Peasants	385 (45.83%)	147 (48.20%)	238 (44.49%)	
Household chores	101 (12.02%)	22 (7.21%)	79 (14.77%)	
*Smoking status*				<0.0001
Nonsmokers	671 (79.88%)	167 (54.75%)	504 (94.21%)	
Current smokers	169 (20.12%)	138 (45.25%)	31 (5.79%)	
*Number of meals per day*				0.7174
<3	217 (25.83%)	81 (26.56%)	136 (25.42%)	
≥3	623 (74.17%)	224 (73.44%)	399 (74.58%)	
*Average walking per day (km)*				<0.0001
<0.5	101 (12.02%)	42 (13.77%)	59 (11.03%)	
0.5–1	305 (36.31%)	86 (28.20%)	219 (40. 93%)	
>1–5	266 (31.67%)	93 (30.49%)	173 (32.34%)	
>5	168 (20.00%)	84 (27.54%)	84 (15.70%)	
*Time used on watching TV (hours)*				0.1035
0	505 (60.62%)	179 (58.88%)	326 (61.63%)	
<2	180 (21.61%)	60 (19.74%)	120 (22.68%)	
≥2	148 (17.77%)	65 (21.38%)	83 (15.69%)	

**Table 2:** Prevalence and odds ratio of abdominal obesity by different selected characteristics of the study population.

Variable	Abdominal obese (%)	OR (95% CI)	*p* value	AOR (95% CI)	*p* value
*Sex*			<0.0001		<0.0001
Men	21 (6.89)	1			
Women	188 (35.14)	7.33 (4.55, 11.81)		8.21 (6.44, 19.52)	
*Marital status*			0.0026		0.0141
Never married	14 (11.97)	1		1	
Married	145 (25.53)	2.52 (1.40, 4.55)		3.31 (1.59, 6.92)	
Divorced/separated	18 (32.14)	3.49 (1.58, 7.69)		3.08 (1.19, 7.96)	
Widowed	32 (32.32)	3.51 (1.75, 7.07)		2.65 (1.08, 6.48)	
*Place of residence*			<0.0001		0.0003
Rural	63 (15.56)	1		1	
Urban	146 (33.56)	2.74 (1.96, 3.83)		2.17 (1.42, 3.31)	
*Age (years)*			0.0004		0.0037
18–30	21 (12.96)	1		1	
31–40	47 (27.81)	2.59 (1.47, 4.57)		2.11 (1.09, 4.08)	
41–50	60 (32.61)	3.25 (1.87, 5.65)		3.42 (1.72, 6.83)	
51+	81 (24.92)	2.23 (1.32, 3.76)		3.00 (1.53, 5.88)	
*Education level*			0.0651		0.0154
Never	28 (20.44)	1		1	
Primary education	120 (23.76)	1.21 (0.76, 1.93)		1.33 (0.78, 2.29)	
Secondary or higher	61 (30.81)	1.73 (1.04, 2.90)		2.57 (1.29, 5.11)	
*Occupation*			0.0021	—	—
Employed	100 (31.35)	1		—	—
Students	4 (11.43)	0.28 (0.10, 0.82)		—	—
Peasant	78 (20.26)	0.56 (0.40, 0.78)		—	—
Household chores	27 (26.73)	0.80 (0.49, 1.32)		—	—
*Smoking status*			<0.0001	—	—
Nonsmokers	196 (29.21)	1		—	—
Current smokers	13 (7.69)	0.20 (0.11, 0.36)		—	—
*Number of meals per day*			0.1459	—	—
<3	46 (21.20)	1		—	—
≥3	163 (26.16)	1.32 (0.91, 1.91)		—	—
*Average walking per day (km)*			0.0371	—	—
<0.5	32 (31.68)	1.79 (1.12, 2.88)		—	—
0.5–1	83 (27.21)	2.22 (1.25, 3.97)		—	—
>1–5	65 (24.44)	1.55 (0.95, 2.53)		—	—
>5	29 (17.26)	1		—	—
*Time used on watching TV (hours)*			<0.0001		0.0165
None	94 (18.61)	1		1	
<2	62 (36.69)	2.53 (1.72, 3.72)		1.99 (1.23, 3.24)	
≥2	41 (27.70)	1.68 (1.10, 2.56)		1.63 (1.03, 2.94)	

## Data Availability

The data used to support the findings of this study are available from the corresponding author upon request.

## Abbreviations

IDF: International Diabetes Federation
NCDs: Noncommunicable diseases
SSA: Sub-Saharan Africa
NBS: National Bureau of Statistics
WHO: World Health Organization.
